# A Systematic Evaluation of Semispecific Peptide Search Parameter Enables Identification of Previously Undescribed N-Terminal Peptides and Conserved Proteolytic Processing in Cancer Cell Lines

**DOI:** 10.3390/proteomes9020026

**Published:** 2021-05-25

**Authors:** Matthias Fahrner, Lucas Kook, Klemens Fröhlich, Martin L. Biniossek, Oliver Schilling

**Affiliations:** 1 Institute for Surgical Pathology, Medical Center–University of Freiburg, Faculty of Medicine, University of Freiburg, 79106 Freiburg, Germany; matthias.fahrner@uniklinik-freiburg.de (M.F.); klemens.erwin.froehlich@uniklinik-freiburg.de (K.F.); 2 Faculty of Biology, Albert-Ludwigs-University Freiburg, 79104 Freiburg, Germany; 3 Spemann Graduate School of Biology and Medicine (SGBM), University of Freiburg, 79104 Freiburg, Germany; 4 Epidemiology, Biostatistics & Prevention Institute, University of Zurich, 8001 Zurich, Switzerland; lucasheinrich.kook@uzh.ch; 5 Institute for Data Analysis and Process Design, Zurich University of Applied Sciences, 8401 Winterthur, Switzerland; 6 Institute for Molecular Medicine and Cell Research, University of Freiburg, 79104 Freiburg, Germany; martin.biniossek@mol-med.uni-freiburg.de; 7 German Cancer Consortium (DKTK) and German Cancer Research Center (DKFZ), 69120 Heidelberg, Germany; 8 BIOSS Centre for Biological Signaling Studies, University of Freiburg, 79104 Freiburg, Germany

**Keywords:** endogenous proteolysis, fragment mass tolerance, mass spectrometry, NCI-60 reanalysis, semispecific peptide search

## Abstract

Liquid chromatography-tandem mass spectrometry (LC-MS/MS) has become the most commonly used technique in explorative proteomic research. A variety of open-source tools for peptide-spectrum matching have become available. Most analyses of explorative MS data are performed using conventional settings, such as fully specific enzymatic constraints. Here we evaluated the impact of the fragment mass tolerance in combination with the enzymatic constraints on the performance of three search engines. Three open-source search engines (Myrimatch, X! Tandem, and MSGF+) were evaluated concerning the suitability in semi- and unspecific searches as well as the importance of accurate fragment mass spectra in non-specific peptide searches. We then performed a semispecific reanalysis of the published NCI-60 deep proteome data applying the most suited parameters. Semi- and unspecific LC-MS/MS data analyses particularly benefit from accurate fragment mass spectra while this effect is less pronounced for conventional, fully specific peptide-spectrum matching. Search speed differed notably between the three search engines for semi- and non-specific peptide-spectrum matching. Semispecific reanalysis of NCI-60 proteome data revealed hundreds of previously undescribed N-terminal peptides, including cases of proteolytic processing or likely alternative translation start sites, some of which were ubiquitously present in all cell lines of the reanalyzed panel. Highly accurate MS2 fragment data in combination with modern open-source search algorithms enable the confident identification of semispecific peptides from large proteomic datasets. The identification of previously undescribed N-terminal peptides in published studies highlights the potential of future reanalysis and data mining in proteomic datasets.

## 1. Introduction

Liquid chromatography-tandem mass spectrometry (LC-MS/MS) has become a well-established method for the explorative and targeted analysis of proteins [1]. In single LC-MS/MS measurements, thousands of peptides and proteins can be identified and quantified. Thus, LC-MS/MS-based proteomics has greatly contributed to the comprehensive investigation of protein alteration on a variety of clinical samples [2,3,4]. The most frequently used method in explorative LC-MS/MS-based proteomics relies on a proteolytic digestion step to cleave proteins into smaller peptides using proteases with known specificities, most often trypsin. In data-dependent acquisition (DDA), peptide identification relies on the precise measurement of the ionized peptide (MS1 level) and the precise measurement of its fragments (e.g., generated by collision-induced dissociation, CID) on the MS2 level. More complex strategies involving MS^n^ are emerging.

A variety of search engines has been developed allowing for the automated and fast identification of peptides by peptide-spectrum matching (PSM) in DDA data. Typically, modifications and digestion enzyme specificity yield a list of possible sequences from a database for a spectrum within a given MS1 accuracy. The experimentally determined fragment masses are then mapped to in silico generated MS2 masses of the candidate sequences, yielding software-specific scores. The inclusion of decoy sequences (typically reverted protein sequences) facilitates the translation of software-specific PSM scores into peptide identifications with defined false discovery rates (FDR). Peptide identifications with an FDR < 1% (less frequently 5%) are deemed reliable in most publications. 

Multiple comparisons of different mass spectrometry search engines as well as search parameters have been published [5,6,7,8]. Most studies focus on the total number of identifications using conventional search settings (e.g., full enzymatic constraints; one or two missed cleavages, relatively narrow mass tolerances).

Conservative peptide searches assume fully specific digestion to minimize the search space. The identification process is highly dependent on the specificity and activity of the selected enzyme in the experimental set-up. Therefore, peptide searches can be further adjusted by including missed cleavages during the protein digestion step. Depending on the number of missed cleavages and the accepted range of peptide lengths (e.g., 600–4000 Da) a fully tryptic search of a human protein database containing 20,000 protein entries covers about 600,000 peptides. However, the search space of a semispecific search with an equal mass range and the same number of entries exceeds 9.0 million non-redundant peptide sequences. Further typical refinements of peptides searches are the inclusion of modifications that might occur on selected amino acids either endogenous or during experimental conditions such as oxidation of methionine. In combination with the addition of decoy sequences, this ultimately leads to increased search spaces. Larger search spaces entail longer analysis time, as well as more stringent cut-off scores using the FDR approach. Consequently, most peptide searches are performed with conservative settings, allowing only the most relevant alterations such as one or two missed cleavages, assuming full enzyme specificity and only the most common variable modifications.

Semispecific peptide searches enable the observation of limited endogenous proteolytic processing. Due to endogenous proteolytic events, proteins are cleaved and truncated proteins are generated. These truncated forms show potentially different terminal amino acids as compared to the specificity of the selected enzyme during the proteomic sample processing. Thus, endogenous N-terminal or C-terminal processing can be observed through the identification of semispecific peptides. Those peptides harbor one cleavage site from endogenous proteases and one known terminal amino acid from the digestion with the specific enzyme during sample processing [9]. Endogenous proteolytic processing is an irreversible post-translational modification, often altering and directly influencing a protein’s role within cellular signaling and response [10]. There has been an extensive effort in protocols allowing for the enrichment and detailed analysis of the endogenous proteolytic processing [11,12,13,14,15,16]. 

The combined effect of less stringent enzymatic constraints and larger fragment mass tolerances has been rarely investigated. Although larger fragment mass tolerances are always assumed to negatively affect the analysis of high-resolution MS data, this has never been tested in the context of semi- and unspecific PSM strategies. Here we used biological samples of different complexity for a qualitative assessment of the performance of three different open-source search engines applying unconventional peptide search parameter settings. We show the expected negative impact of increasing mass tolerances in the analysis of high-resolution MS data. Interestingly, we show a synergistic negative effect of larger fragment mass tolerances in combination with less stringent enzymatic constraints such as semi- and unspecific searches. Published data provides a valuable and often untapped resource for reanalysis using unconventional peptide searches. We performed a semispecific peptide search on the published deep proteome data of the NCI-60 project and identified previously undescribed protein N-termini [17].

## 2. Materials and Methods

### 2.1. Proteomic Sample Preparation of the Different Specimens

#### 2.1.1. Murine Formalin-Fixed, Paraffin-Embedded FFPE Kidney Tissue 

Three adjacent slides of murine formalin-fixed, paraffin-embedded (FFPE) kidney samples were prepared as previously described using the filter-aided sample preparation protocol (FASP) [18] except for the enzymatic digestion. Here, 2 μg of chymotrypsin was added to each sample and incubated at 37 °C overnight, followed by adding another 2 μg of fresh chymotrypsin to each sample and incubating at 50 °C for 3 h. The peptide concentration was measured using a bicinchoninic acid (BCA) assay and 2 μg of peptides were vacuum-dried until mass spectrometry measurement.

#### 2.1.2. Human FFPE Liver Metastasis Sample

Three adjacent slides of a human FFPE liver metastasis sample were prepared using the previously described direct tissue trypsinization (DTR) protocol [18] except for the enzymatic digestion step. Here, the proteins were digested by adding 2 μg of LysC to each sample and incubating for 3 h at 50 °C. Subsequently, samples were allowed to cool down to room temperature and 4 μg of Trypsin was added, followed by incubating at 37 °C overnight. Peptides were cleaned up using Hypsersep C18 tips (Thermo Scientific, Waltham, MA, USA) according to the manufacturer’s protocol.

#### 2.1.3. Human Embryonic Kidney (HEK293T) Whole Proteome Samples

Four replicates of HEK cells were washed three times with phosphate-buffered saline (PBS) and subsequently lysed in 100 mM 4-(2-hydroxyethyl)-1-piperazineethanesulfonic acid (HEPES) pH 8.5, containing 0.1% RapiGest. Samples were denatured and reduced at 95 °C for 10 min using 10 mM tris(2-carboxyethyl)phosphine (TCEP) and DNA was sheared using a Bioruptor. Afterward, 20 mM iodoacetamide was added and samples were incubated for 30 min in the dark at room temperature. 100 μg of lysate were digested at 37 °C overnight, using 2 μg of sequencing grade LysC. RapiGest was degraded and peptides were cleaned, using the STAGE-TIP protocol [19] and vacuum-dried until MS measurement.

### 2.2. LC-MS/MS Analysis

LC-MS/MS measurements were performed as previously described [18] on an Orbitrap Q-Exactive plus (Thermo Scientific) mass spectrometer coupled to an Easy nanoLC 1000 (Thermo Scientific). Briefly, high-resolution mass spectrometry data was acquired using the data-dependent acquisition mode. First, a precursor scan covering the mass-to-charge range from 300 to 2000 m/z at 70,000 resolution was performed in profile mode. Subsequently, a series of fragmentation scans of up to 10 most intense precursors at 17,500 resolution was acquired in centroided mode.

### 2.3. Data Analysis Using OpenMS

Raw data were converted to the open file format mzML using msconvert [20] (ProteoWizard 3.0.10386) using the default settings with the additional filter setting “metadataFixer”. An analysis workflow for the three different search engines was generated using OpenMS [21] (v 2.3) and the TOPPAS [22] workflow editor (Supplementary Figure S1). Three different search engines were employed using OpenMS adapter: Myrimatch (v2014.07.04), X! TANDEM Alanine (2017.2.1.4) and MSGF+ (v2017.08.23). Evaluated parameter settings were iterated in a fully automated manner using a windows batch script modifying single parameters for every peptide search run. All searches were performed allowing for oxidation (M) and acetylation (Protein-N-term) as variable modification and defining a precursor mass tolerance of 10 ppm. In X! Tandem and Myrimatch, one missed cleavage was allowed and searches were iterated using different enzymatic constraints as well as fragment mass tolerances. In MSGF+ all searches were performed using high-resolution fragment mass tolerance “HighRes” and were iterated for different enzymatic constraints. All peptide searches analyzing the murine FFPE samples were performed using a reviewed database (UniProt, June 2017) containing 17,126 entries. For chymotrypsin the default specificity rules were employed. Of note, these vary slightly between the different search engines: cleavage after FWYL also before P for X! Tandem and MSGF+; cleavage after FWYLI but not before P for Myrimatch. For the analysis of the HEK cell proteome and the human FFPE samples, a reviewed database (UniProt, June 2017) containing 20,445 entries was used. All search results were filtered for 5% FDR on the PSM level. Search results were further processed using R (v 3.6.0) in RStudio (Version 1.1.456) and visualized using the R package ggplot2 (v 3.2.1). Decoy identification, peptides originating from contaminants as well as ambiguous peptide sequences mapping to multiple proteins were removed. Unique peptides which were identified multiple times (e.g., carrying variable modifications as well as unmodified) were further filtered based on their respective sequence yielding total numbers of non-redundant peptide hits for each replicate in each search. The elapsed time for the complete analysis of all replicates with the shown workflow was recorded (Supplementary Figure S1).

### 2.4. Reanalysis of Published NCI-60 Data

Deep proteome data from nine representative cancer cell lines were retrieved from https://www.proteomicsdb.org/proteomicsdb/#projects/35 (1 August 2018) [17]. The authors applied molecular weight gel-based separation on the cell lysates before the tryptic digestion. After desalting the peptides were measured on an LTQ Orbitrap Elite mass spectrometer using data-dependent acquisition applying the orbitrap mass analyzer for the MS1 and the MS2 scans yielding high-resolution mass spectrometry data. The NCI-60 deep proteome raw data was converted using msconvert using default settings except for applying the “metadataFixer” filter. Files were analyzed using the workflow manager web interface Galaxy (https://usegalaxy.eu) [23] and the implemented OpenMS tools (v 2.3). The search engine MSGF+ was used with 10 ppm precursor mass tolerance, semitryptic enzymatic constraint, fixed carbamidomethyl modification as well as oxidation (M) and acetylation (Protein-N-term) as variable modification. The instrument type was set to “Q_Exactive” applying low fragment mass tolerance since the data was generated using high-resolution MS. A reviewed human protein database containing 20.259 entries (UniProt) was used. Additionally, reversed and shuffled decoy sequences were added to the database, enabling confident peptide identification based on 1% FDR on the PSM level. Comprehensive analysis histories of all nine peptide searches are shared and can be accessed via galaxy. Preliminary peptide identification results were filtered for unique peptides, matching only one protein entry as well as peptides that were identified in at least two out of the nine cell lines yielding a subset of more confident peptide identifications. Peptides originating from either the N-terminal removal of methionine or the native protein C-terminus were excluded. Peptide identifications were filtered for fully tryptic peptides, that were identified in at least five of the nine cell lines and their respective proteins. The molecular weight (MW) of those stably identified proteins was log2 transformed and the median MW per gel slice was calculated (Supplementary Figure S2, exemplarily shown for CCRF-CEM cells). Using linear models, a linear regression of the median MW of identified proteins and the respective gel slice was computed for each of the nine cell lines. Next, the preliminary peptide identifications were filtered for N-terminal semispecific peptides excluding fully tryptic and C-terminal semispecific peptides. The linear regression fit based on the tryptic peptides was applied to compute expected gel slices of the proteins associated with the N-terminal semitryptic peptides. Briefly, the length of the N-terminally truncated protein was calculated, and its MW was estimated with an average MW of 100 Da per amino acid residue. The N-terminal semitryptic peptides were further filtered for peptides originating from proteins where the expected gel slice was the same or directly adjacent to the observed gel slice. The remaining peptides were considered as confidently identified semitryptic N-terminal peptides. Proteins for which at least 10 N-terminal peptides were identified in one of the cell lines and for which peptides were missing in at most one of the cell lines were defined as hotspots of endogenous proteolytic events. Identified N-terminal peptides were filtered for peptides occurring in all of the nine cancer cell lines.

## 3. Results

### 3.1. Reduced Enzymatic Constraints Combined with Increased Fragment Mass Tolerances Lead to Fewer Peptide Identifications in High-Resolution LC-MS/MS (MS) Data

**Figure 1:**
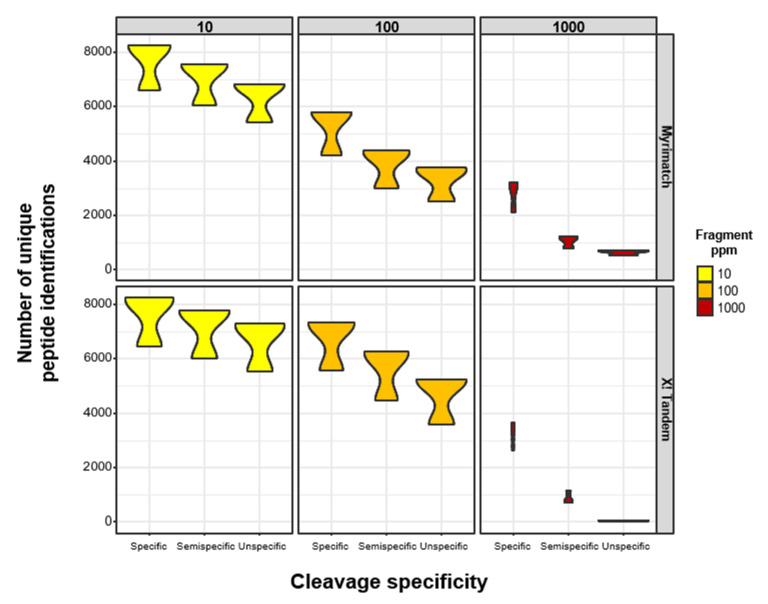
Effect of less stringent enzymatic constraints and fragment mass tolerances on peptide identification results. Human formalin-fixed, paraffin-embedded (FFPE) samples were digested using LysC and Trypsin and were analyzed using Myrimatch (upper panel) and X! Tandem (lower panel). The number of identified unique non-redundant peptide identifications of the three replicates are shown in a violin plot according to the enzymatic constraint and the fragment mass tolerance (10, 100, and 1000 ppm) of the search engine settings.

We analyzed three replicates of a human liver metastasis sample to investigate the impact of the less stringent enzymatic constraints in combination with different fragment mass tolerances on the number of peptide identifications. The tissue was formalin-fixed and paraffin-embedded (FFPE), representing a realistic biological sample used in quantitative proteomic studies [24]. Data acquisition was performed using an orbitrap mass analyzer yielding high-resolution mass spectrometry data on both the precursor (MS1) as well as the fragment (MS2) level. Two open-source search engines were applied, both allowing to freely define the fragment mass tolerance as well as the enzymatic specificity. We identify between 6000–8000 non-redundant peptides using a narrow fragment mass tolerance of 10 ppm with X! Tandem and Myrimatch (Figure 1). For both search engines, the number of identified peptides decreases with a less specific enzymatic search setting. Interestingly, the difference in the number of identified peptides between the enzymatic specificities increases for larger fragment mass tolerances. Consequently, when 1000 ppm fragment mass tolerance is set the number of identified peptides ranges from 0 to over 3000 between the different enzymatic constraints. As expected, a higher MS2 mass tolerance leads to decreased number of peptide identifications in the analysis of high-resolution MS data. However, a negative synergistic effect of the enzymatic constraints and the fragment mass tolerance setting in the analysis of human FFPE tissue can be observed. Thus, emphasizing the importance of accurate MS2 data, especially for non-conventional PSM strategies. Consequently, high-resolution MS2 data and narrow mass tolerances seem to be required for semi and unspecific peptide searches.

### 3.2. Semispecific PSMs Yield Comparable Numbers of Peptide Identifications Using Different Open-Source Search Engines

**Figure 2:**
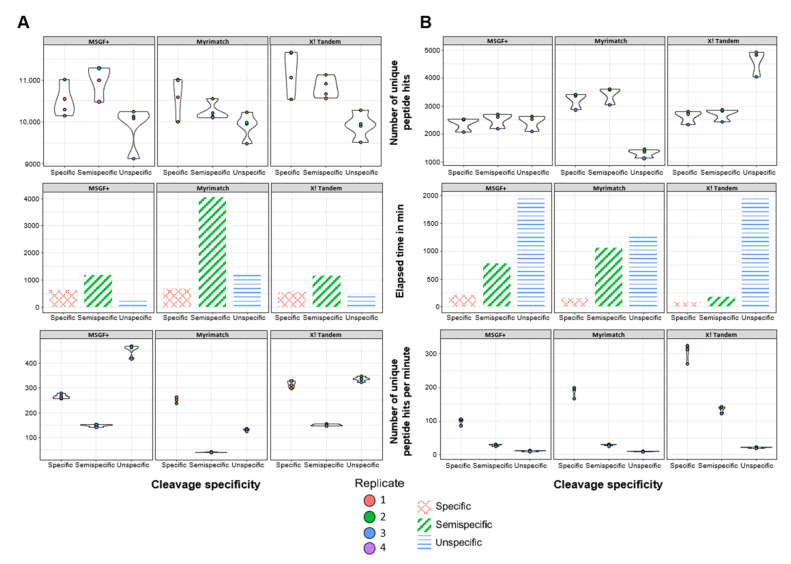
Peptide search results from three different open-source search engines. Four biological replicates of Human Embryonic Kidney (HEK293T) cell proteome (**A**) and three adjacent formalin-fixed, paraffin-embedded (FFPE) tissue slides of Murine kidney (**B**) samples were digested using either LysC (**A**) or chymotrypsin (**B**) and were analyzed using MSGF+ (left), Myrimatch (middle), and X! Tandem (right). The number of identified unique non-redundant peptide hits (upper panel), the elapsed analysis time in min (middle panel) as well as the number of identified unique peptides per time (lower panel) is illustrated according to the enzymatic constraint settings.

To investigate the potential benefit of semi and unspecific peptide searches we analyzed four biological replicates of HEK proteome samples digested with LysC. For this analysis, we also applied MSGF+ in addition to X! Tandem and Myrimatch. All three open-source search engines were used with narrow mass tolerances for both the MS1 as well as the MS2 level. The number of identified peptides using specific and semispecific enzymatic specificity are comparable for all three search engines and range between 10,000–11,000 unique peptide identifications (Figure 2A, upper panel). The unspecific searches yield lower peptide identifications for all three search engines ranging from 9000–10,000 unique peptides, which is most likely caused by the exponential increase in the search space while keeping the overall FDR at 5% on the PSM level. Noteworthy, applying a more stringent FDR of 0.5% on the PSM level yields lower numbers of peptide identifications while showing similar identification profiles when comparing the different cleavage specificities (shown for MSGF+ in Supplementary Figure S3). The analysis times for fully specific searches are comparable between the different search engines, whereas the analysis times widely differ for the semi- and unspecific searches (Figure 2A, middle panel). Noteworthy, the peptide searches with semispecific enzymatic specificity are the most time-consuming for all three search engines. A combined investigation of the number of identified peptides and the elapsed analysis times highlights the benefits of specific searches compared to semispecific searches in the analysis of the HEK proteome samples (Figure 2A, lower panel).

We further assessed the impact of less stringent enzymatic constraints investigating murine FFPE kidney tissue. Performing LysC or Trypsin digestion results in positively charged peptides at their C-terminus, which can favor y-ion generation during gas-phase fragmentation. To test the ability of the search engines to identify peptides potentially lacking C-terminal basic residues, samples were prepared using chymotrypsin, which we consider to be a rather loosely specific protease. In general, all searches yielded lower numbers of identified peptides compared to the human liver metastasis samples prepared with trypsinization and the HEK samples prepared with LysC (Figure 2B, upper panel). The three search engines identify between 2000–3500 non-redundant peptides in the specific and semispecific searches. A systematic decrease of peptide identifications with less stringent enzymatic constraints is not observable in the murine FFPE samples. However, for the unspecific peptide searches, the number of identified peptides widely differs among the search engines yielding between 1500 (Myrimatch) and 4500 (X! Tandem) non-redundant peptide identifications. As expected, due to the increased search space, the analysis time increased notably with less stringent enzymatic constraints for all three search engines. For instance, the elapsed time for the analysis of three replicates increased 10-fold comparing specific and unspecific searches using MSGF+ and X! Tandem (Figure 2B, middle panel). Considering the number of non-redundant peptide identifications in combination with the elapsed analysis time a clear benefit of the specific and semispecific searches as compared to the unspecific searches is observable (Figure 2B, lower panel). 

### 3.3. Semispecific Reanalysis of Published Data Enables the Identification of Previously Non-Described N-Terminal Peptides

Reanalysis focusing on semitryptic peptides has been shown to yield novel insights, providing more comprehensive data on endogenous biological processes in tissue proteomic studies [25]. Of the three different search engines, MSGF+ yields higher numbers of identified peptides for semispecific searches as compared to the fully specific searches for both datasets (Figure 2). We opted to use MSGF+ for the semispecific reanalysis of published data, as we observed more presently ongoing software development as compared to the Myrimatch and X! Tandem software. However, required analysis time and computational resources can be challenging due to the increased search spaces of semi- and unspecific searches, as highlighted for the different datasets (Figure 2). 

**Figure 3:**
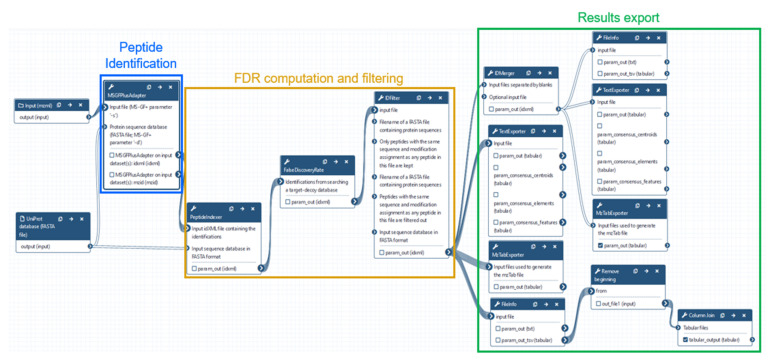
Large-scale semispecific reanalysis of published NCI-60 deep proteome dataset. Workflow used for the analysis of published NCI-60 deep proteome data using OpenMS tools in a workflow within the Galaxy framework. Whole proteome samples of nine representative cancer cell lines were separated into 24 samples using gel-based molecular weight separation. Peptide identification was performed using MSGF+ with semitryptic enzymatic constraint, followed by false discovery rate (FDR) computation and filtering for 1% FDR on the peptide-spectrum matching (PSM) level.

To provide the whole field the opportunity to harness the potential benefits of semispecific searches without the local restrictions of computational resources and the investment therein, we assembled a robust semispecific reanalysis pipeline using the European Galaxy server. The workflow is available at https://usegalaxy.eu/u/matthiasfahrner/w/msgf-23-semispecific-peptide-identification-nci-60-data (Figure 3). 

**Figure 4:**
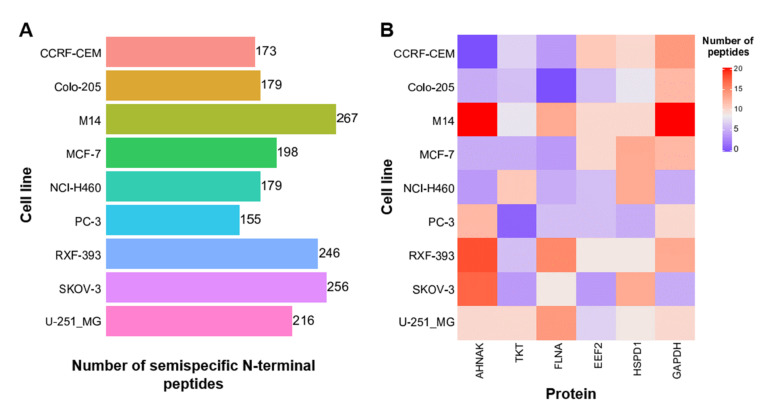
Identification of semispecific N-terminal peptides and proteins with prominent endogenous proteolytic processing. (**A**) Bar chart showing the number of confidently identified semispecific N-terminal peptides. Primary identification results from semispecific peptide searches were filtered for unique peptides, which were identified in at least two out of nine cell lines. Peptides originating from protein N-terminal methionine clipping or representing the native C-terminus were excluded. Only semispecific N-terminal peptides derived from proteins that were proximal to the expected molecular weight gel slice were considered (Supplementary Figure S2). (**B**) Heatmap showing proteins for which at least 10 peptides were identified in at least one of the nine cell lines. The color indicates the number of semispecific peptides identified per protein in the respective cell lines.

Within Galaxy, we used OpenMS software for a high-throughput semispecific reanalysis of the NCI-60 deep proteome dataset. In this present, publicly deposited Galaxy workflow, we primarily focused on semitryptic peptide identifications as a measure of endogenous limited proteolytic processing. As for any Galaxy workflow, users can derive a customizable copy of this workflow—e.g., to include variable modification in the peptide identification process as a measure of PTMs in combination with a semispecific peptide identification. The Galaxy implementation of the search engine has access to the spectrum of modifications as defined by the UniMod database; in this particular case as integrated into OpenMS version 2.3. In the original project, the authors performed gel-based molecular weight separation of the proteomes of nine different human cancer cell lines [26]. In our reanalysis pipeline, we focus on unique peptides, which are specific for one protein, and on peptides that were identified in at least two out of the nine cell lines. Peptides derived from the removal of protein N-terminal methionine were excluded. For simplicity, we focused on peptides with a non-tryptic N-termini. Furthermore, the molecular weight information from the gel-based separation was used to select peptides for which the endogenously cleaved protein was approximately close to the expected gel slice (Supplementary Figure S2). After refinement of the peptide identification results, the semispecific searches yield between 155 and 267 confident semispecific N-terminal peptides in each of the nine cell lines (Figure 4A).

Combined analysis of the confidently identified semitryptic peptides reveals six proteins for which more than 10 semitryptic peptides were identified in at least one of the nine cell lines (Figure 4B). For the housekeeping gene GAPDH, 20 semitryptic peptides were identified in the melanoma cell line M14 and over 10 semitryptic peptides in the cell lines CCRF-CEM, Colo-205, MCF-7, and RXF-393. These proteins represent hotspots of endogenous proteolytic events, resulting in multiple semispecific peptides. Furthermore, we focused on “non-conventional” protein N-termini which were ubiquitously identified in all nine cell lines (Table 1) yielding a confined core set of protein N-termini that deviate from the N-termini as defined in the human sequence database which we have employed. For ease of readability, we will refer to these N-termini as neo N-termini. Three neo N-termini are acetylated, suggesting co-translational acetylation. For the protein XPO4, the identified peptide starts at position 3 of the full-length protein, following two methionines. This could be due to a double clipping by methionyl aminopeptidase. For the protein XPO1, the acetylated neo N-terminus is at position 6 of the full-length protein, following a methionine residue, hence likely representing an alternative translation initiation site (ATIS) as previously described [27,28]. For the protein Kanadaptin (NADAP) the neo N-terminus is at position 56 of the full-length protein, following a methionine residue, possibly representing an ATIS. Two neo N-termini (non-acetylated) represent signal peptide cleavage sites (EMC1 and HYOU1). Note, that for HCFC1, the identified semitryptic peptide represents the N-terminal part of the HCF C-terminal chain 4 and has been described to occur due to autolytic cleavage at one or several PPCE—THET motifs within the full-length protein [29].

In total, we identified 12 ubiquitously present neo N-termini in the nine cancer cell lines (Supplementary Table S1). Many more neo N-termini are present in multiple but not all of the reassessed cancer cell lines. Our small-scale study highlights the potential of semispecific reanalysis of published data as well as the need for further investigation of endogenous processing and the biological implications thereof.

## 4. Conclusions

We show the importance of accurate fragment (and of course precursor) mass determination for less stringent enzymatic constraints in peptide-spectrum matching. Furthermore, we illustrate the value of semispecific searches in identifying functional protein N-terminal biology from classical shotgun proteomics dataset without particular enrichment steps. This opens the perspective of novel insights into proteome biology by mere reanalysis of previously published (and publicly deposited!) LC-MS/MS datasets. The potential benefit of unspecific and semispecific searches is highly dependent on the proteases used during the sample preparation and the biological sample. For highly specific proteases such as LysC or trypsin, the completely unspecific searches yield fewer peptide identification and consume considerably more analysis time. However, for broadly specific proteases such as chymotrypsin, the less stringent enzymatic searches provide the opportunity for the identification of more and previously unidentified peptides in high-resolution mass spectrometry data. Depending on the complexity of the biological sample, the increase in peptide and protein identifications with less stringent peptide searches comes with an increase in analysis time. Thus, the potentially larger numbers of identifications are introduced with challenges such as the requirement for vast computational resources and longer analysis time. Using publicly available resources via the galaxy framework, we were able to perform a large-scale semispecific reanalysis of the previously published deep proteomes of nine representative cancer cell lines. The analysis revealed previously unidentified N-terminal peptides as well as proteins reflecting potential hotspots of endogenous proteolytic events. We identified 12 neo N-termini which occurred in all nine cell lines, likely representing conserved biological processes.

## Figures and Tables

**Table 1:** List of conserved N-terminal peptide sequences identified in nine representative cancer cell lines. The list of confidently identified semitryptic peptides was filtered for peptides that were identified in all nine cell lines (see Figure 4A).

Accessions	Sequence	Function	AA Before	Position	Expected Gel Slice	Observed Gel Slice
sp|Q9C0E2|XPO4_HUMAN	.(Acetyl)AAALGPPEVIAQLENAAK	Double clipping **	M	3	5	6
sp|Q9BWU0|NADAP_HUMAN	.(Acetyl)ADILSQSETLASQDLSGDFKKPALPVSPAAR	Potential ATIS *	M	56	7	7
sp|O14980|XPO1_HUMAN	.(Acetyl)TM(Oxidation)LADHAAR	ATIS *	M	6	6	7
sp|Q9Y4L1|HYOU1_HUMAN	LAVM(Oxidation)SVDLGSESM(Oxidation)K	Removal of signal peptide	T	33	6	6
sp|P51610|HCFC1_HUMAN	THETGTTNTATTSNAGSAQR	Cleavage by autolysis/HCF C-terminal chain 4	E	1296	7	8
sp|Q8N766|EMC1_HUMAN	VYEDQVGK	Removal of signal peptide	A	22	6	7

* ATIS = Alternative translation initiation site. ** Double clipping of methionyl aminopeptidase.

## Data Availability

Mass spectrometry data, as well as data analysis results, have been deposited to ProteomeXchange via MassIVE (ID: PXD024676, https://massive.ucsd.edu/ProteoSAFe/private-dataset.jsp?task=5ddff492950e4083886fab6b623c08fb). The reanalysis results of the NCI-60 deep proteome data are shared as complete galaxy histories (https://usegalaxy.eu/u/matthiasfahrner/h/rxf-393, https://usegalaxy.eu/u/matthiasfahrner/h/colo-205, https://usegalaxy.eu/u/matthiasfahrner/h/pc-3, https://usegalaxy.eu/u/matthiasfahrner/h/nci-h460, https://usegalaxy.eu/u/matthiasfahrner/h/u-251-mg, https://usegalaxy.eu/u/matthiasfahrner/h/skov-3, https://usegalaxy.eu/u/matthiasfahrner/h/m14, https://usegalaxy.eu/u/matthiasfahrner/h/mcf-7, https://usegalaxy.eu/u/matthiasfahrner/h/ccrf-cem).

## Supplementary Materials

The following are available online at https://www.mdpi.com/article/10.3390/proteomes9020026/s1, Figure S1: Peptide Identification workflow in TOPPAS using OpenMS tools; Figure S2: Linear regression analysis of gel-based molecular weight separation in NCI-60 deep proteome samples; Figure S3: Peptide search results filtered for 0.5% FDR at the PSM level; Table S1: Complete list of conserved N-terminal peptides among nine representative cancer cell lines.
