# Supplementary figures and images for: A Systematic Evaluation of Semispecific Peptide Search Parameter Enables Identification of Previously Undescribed N-Terminal Peptides and Conserved Proteolytic Processing in Cancer Cell Lines
Source: Proteomes. 2021 May 25;9(2):26. doi: 10.3390/proteomes9020026 (PMC8162549; doi:10.3390/proteomes9020026)

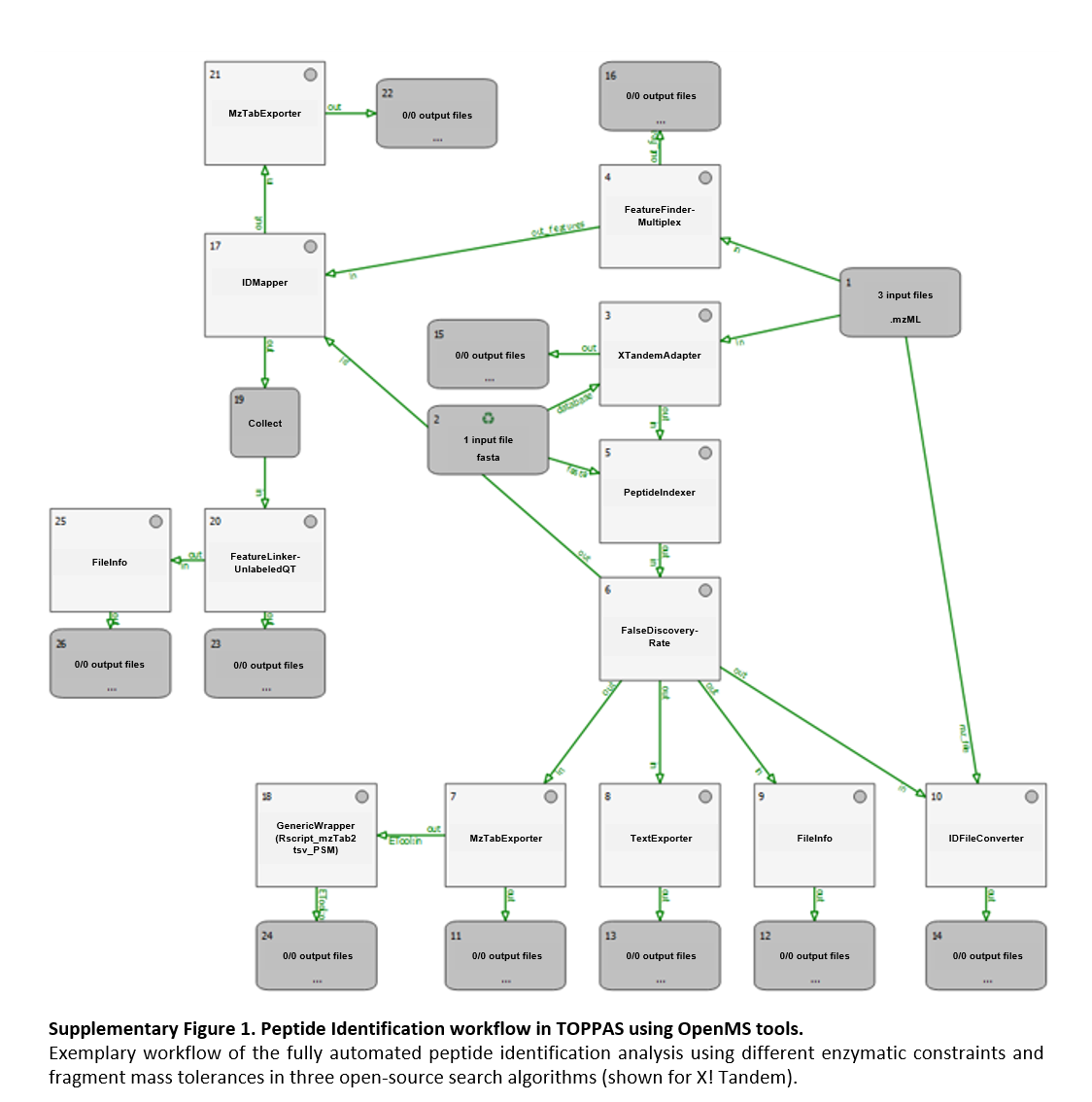

Supplement: Supplementary file 1 [file proteomes-09-00026-s001.zip › Supplementary_Materials/Supp_Figure_S1.png]

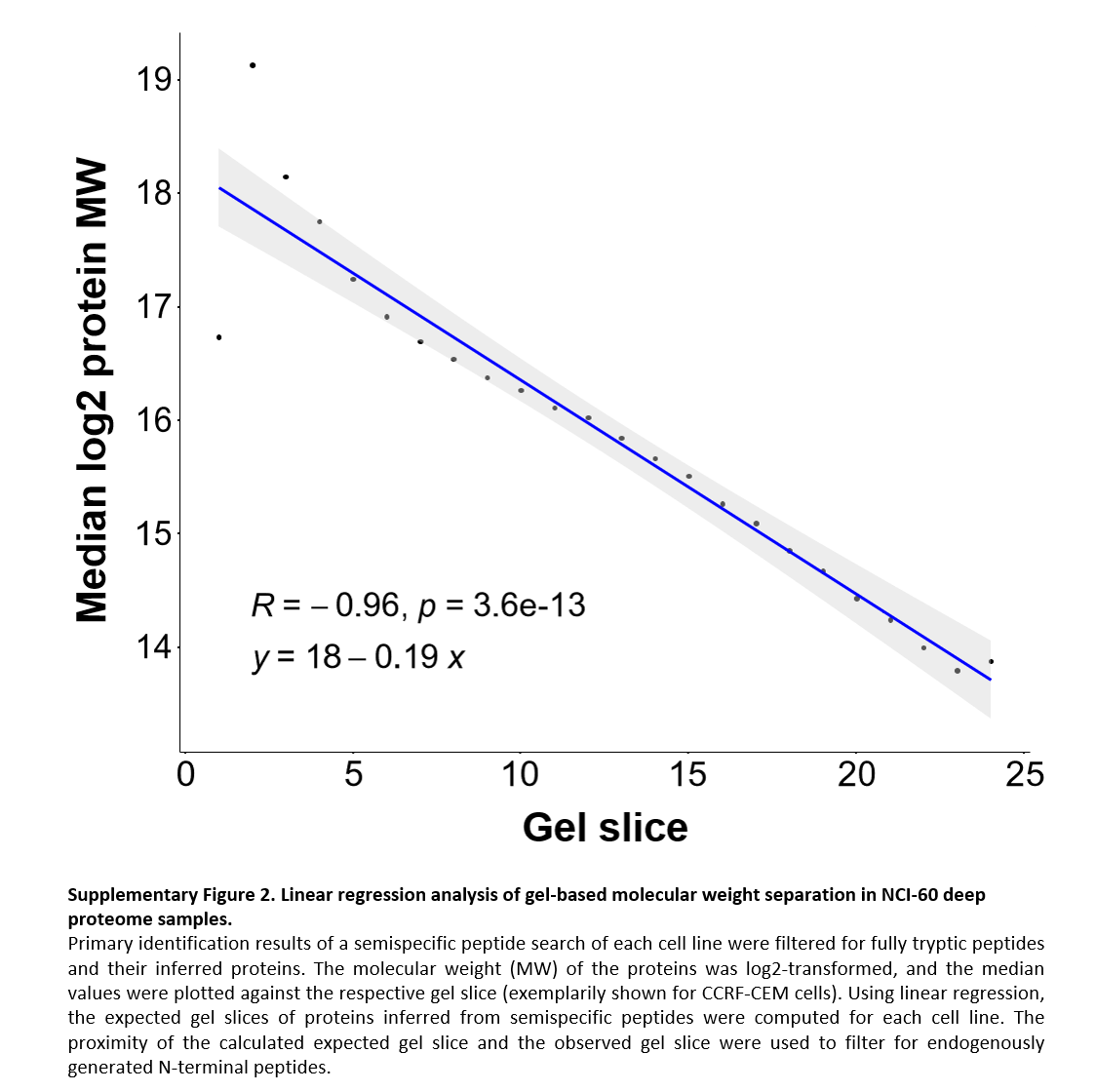

Supplement: Supplementary file 1 [file proteomes-09-00026-s001.zip › Supplementary_Materials/Supp_Figure_S2.png]

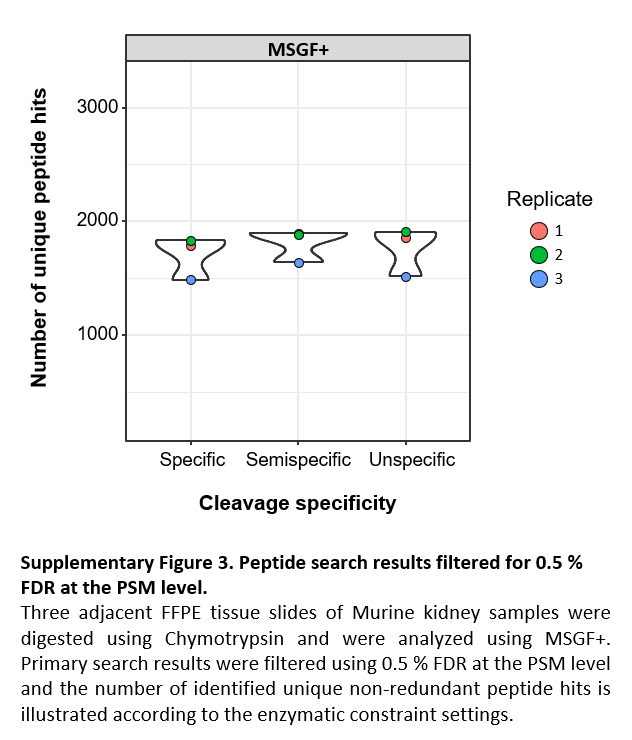

Supplement: Supplementary file 1 [file proteomes-09-00026-s001.zip › Supplementary_Materials/Supp_Figure_S3.png]

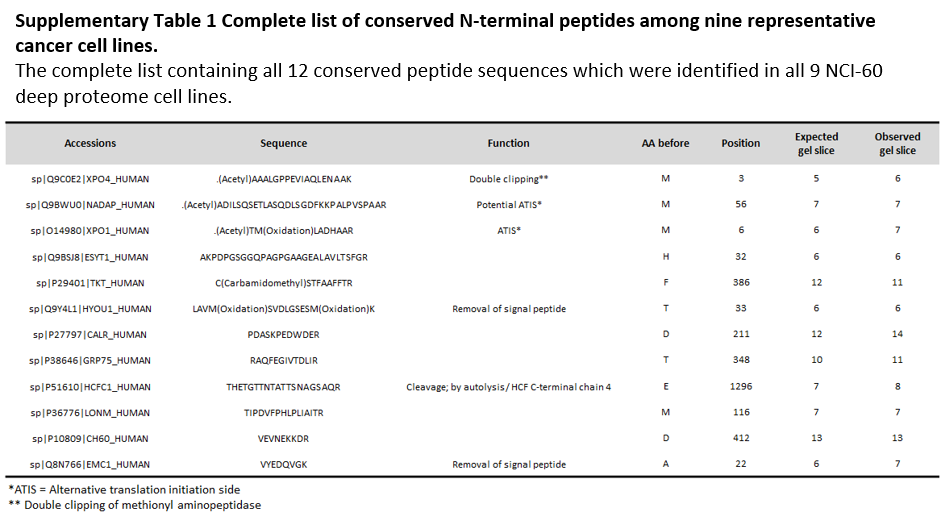

Supplement: Supplementary file 1 [file proteomes-09-00026-s001.zip › Supplementary_Materials/Supp_Table_S1.png]
